# Nuclear nanomedicine using Si nanoparticles as safe and effective carriers of ^188^Re radionuclide for cancer therapy

**DOI:** 10.1038/s41598-018-38474-7

**Published:** 2019-02-14

**Authors:** V. M. Petriev, V. K. Tischenko, A. A. Mikhailovskaya, A. A. Popov, G. Tselikov, I. Zelepukin, S. M. Deyev, A. D. Kaprin, S. Ivanov, V. Yu. Timoshenko, P. N. Prasad, I. N. Zavestovskaya, A. V. Kabashin

**Affiliations:** 1 MEPhI, Institute of Engineering Physics for Biomedicine (PhysBio), 115409 Moscow, Russia; 2 National Medical Research Radiological Center of the Ministry of Health of the Russian Federation, Obninsk, Russia; 3 Aix Marseille Univ, CNRS, LP3, Campus de Luminy – Case 917, 13288 Marseille, France; 4 Shemyakin–Ovchinnikov Institute of Bioorganic Chemistry, Russian Academy of Sciences, 16/10 Miklukho-Maklaya St, Moscow, 117997 Russia; 5 Lomonosov Moscow State University, Physics Department, Leninskie Gory 1, 119991 Moscow, Russia; 6 Department of Chemistry and Institute for Lasers, Photonics, and Biophotonics, University at Buffalo, The State University of New York, Buffalo, New York, 14260 United States; 7 National Research Tomsk Polytechnic University, Tomsk, Russia

## Abstract

Nuclear nanomedicine, with its targeting ability and heavily loading capacity, along with its enhanced retention to avoid rapid clearance as faced with molecular radiopharmaceuticals, provides unique opportunities to treat tumors and metastasis. Despite these promises, this field has seen limited activities, primarily because of a lack of suitable nanocarriers, which are safe, excretable and have favorable pharmacokinetics to efficiently deliver and retain radionuclides in a tumor. Here, we introduce biodegradable laser-synthesized Si nanoparticles having round shape, controllable low-dispersion size, and being free of any toxic impurities, as highly suitable carriers of therapeutic ^188^Re radionuclide. The conjugation of the polyethylene glycol-coated Si nanoparticles with radioactive ^188^Re takes merely 1 hour, compared to its half-life of 17 hours. When intravenously administered in a Wistar rat model, the conjugates demonstrate free circulation in the blood stream to reach all organs and target tumors, which is radically in contrast with that of the ^188^Re salt that mostly accumulates in the thyroid gland. We also show that the nanoparticles ensure excellent retention of ^188^Re in tumor, not possible with the salt, which enables one to maximize the therapeutic effect, as well as exhibit a complete time-delayed conjugate bioelimination. Finally, our tests on rat survival demonstrate excellent therapeutic effect (72% survival compared to 0% of the control group). Combined with a series of imaging and therapeutic functionalities based on unique intrinsic properties of Si nanoparticles, the proposed biodegradable complex promises a major advancement in nuclear nanomedicine.

## Introduction

Сancer therapy using radiopharmaceutical products has become increasingly important over the last decades, promising an attractive and powerful alternative to conventional chemotherapy^1^. This nuclear medicine modality implies an injection of short decay time radionuclides *in vivo* (systemically or intratumorally), while their ionizing radiation (α, β, γ) is used to damage the DNAs of actively proliferating cancer cells, thus causing their selective death while keeping normal cells weakly affected^1^. The radionuclide therapy becomes especially efficient when one can achieve a high tumor/non-tumor radionuclide contrast, which enables to minimize side effects related to the irradiation of healthy issues. In a conventional approach, one employs vectoring molecules (specific antibodies, etc.) to target radionuclides to the tumor, but these molecules are typically small (less than 60–65 kDa) and can carry only a few chelates linked to radionuclide atoms^2,3^. Consequently, one has to deliver very high concentrations of radionuclide-carrying molecules to achieve any sufficient therapeutic effect, but this leads to severe side effects, taking into account that the efficiency of molecular targeting typically does not exceed 10–12%. In addition, the size of most targeting molecules appears to be within the renal glomerular filtration range (<7 nm)^4^, which leads to too fast accumulation of radionuclide complexes in the kidney, causing consequent interstitial nephritis or renal failure problems^5,6^.

Recently, there has been a great deal of interest in developing nuclear nanomedicine which utilizes nanoparticles (NPs) as carriers of radionuclides^7,8^. When functionalized by biopolymers such as polyethylene glycol (PEG), NPs promise safe and controllable transport of radionuclides in the blood stream, as well as offer a passive vectoring mechanism for targeting tumors based on their selective size accumulation (enhanced permeability and retention (EPR) effect)^2^. In addition, NPs can be more heavily loaded with radionuclides to ensure an enhanced therapeutic outcome in the tumor region^7,8^. However, some stringent requirements to make nuclear medicine safe and effective, have been challenging. The challenges to be met are: (1) NPs-based carrier should be large enough (>20–30 nm) to avoid immediate renal filtration and ensure efficient delivery of radionuclides to the intended site; (II) the NP –radiopharmaceutical conjugate should be safe and excretable from the organism to minimize toxicity and residual accumulation risks^4,9^; (III) the NP –conjugate should have targeting ability to effectively localize in high concentrations in the tumor; (IV) the coupling to the radioactive nuclei should be fast compared to their half life in order to maximize radiation therapy. Despite the presence of several classes of highly biocompatible nanomaterials, these challenges are very difficult to meet, as the required large size of NPs beyond the renal filtration range drastically complicates their further bioelimination^4,10^.

In this article, we propose a pathway to meet these challenges by introducing silicon (Si) NPs (Si*NPs), synthesized by pulsed laser ablation in liquids^11–13^, as a nearly ideal carrier of radionuclides for nuclear nanomedicine. The uniqueness of such Si*NPs is based on their biodegradability, which makes possible elimination of these structures from the organism within several days, even if their initial size is large (30–80 nm)^12,13^ under absence of any toxic effects, which was earlier confirmed in a mice model^12^. In addition, in contrast to Si nanostructures prepared by conventional electrochemical^14^ or chemical^15^ routes, laser-synthesized Si*NPs have ideal round shape, controllable size with a small size dispersion, and are free of any toxic impurities^11^, which promises a better transport *in vivo* and no side effects. Here, we demonstrate the possibility for coating of laser-synthesized Si*NPs by PEG and a fast conjugation of the Si*NPs-PEG complex with the Rhenium-188 (^188^Re) radionuclide, which is one of most promising generator-type therapeutic beta-emitters with the energy of positron emission of 1.96 MeV (16.7%) and 2.18 MeV (80%) and half-decay time of 17 hours^1^. We show that such Si*NPs-PEG-^188^Re conjugates can efficiently deliver the radionuclide through the blood stream and retain it in the tumor region. We also demonstrate strong therapeutic effect under intratumoral administration of the conjugate.

## Results and Discussion

### Fabrication, characterization and functionalization of Si*NPs

**Figure 1:**
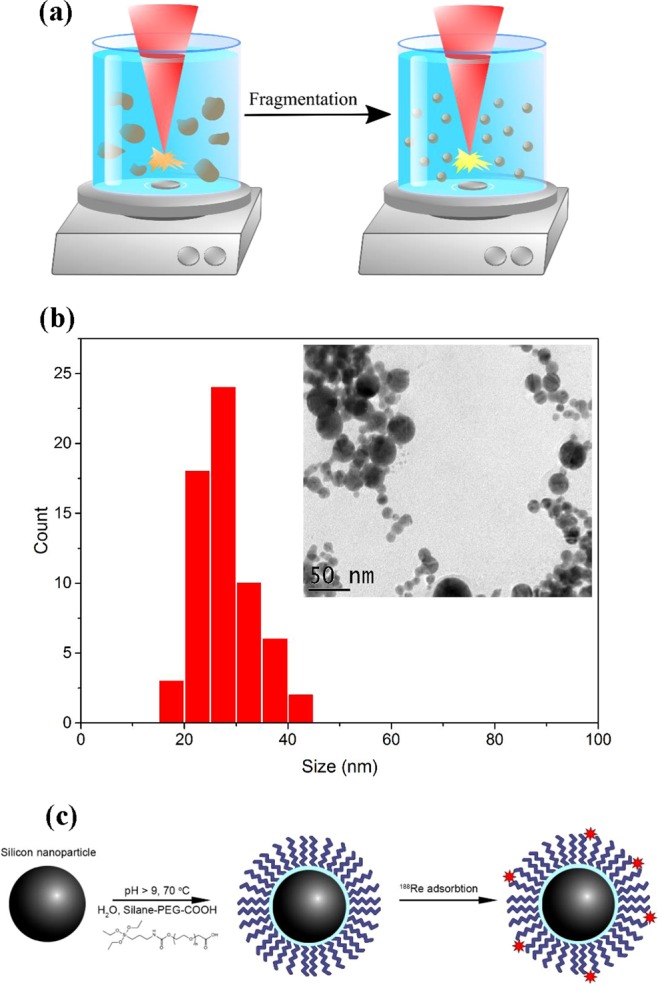
Synthesis and functionalization of Si nanoparticles for nuclear medicine tasks. (**a**) Schematic of laser synthesis of Si*NPs. Crystalline Si microcolloids (~0.5 μm in size), preliminarily prepared by mechanical milling of a Si wafer, are dispersed in deionized water and illuminated by focused radiation from fs laser. The laser-ablative process leads to the formation of spherical, small size-dispersed Si*NPs exempt of any toxic impurity; (**b**) Typical transmission electron microscopy image (inset) and corresponding size distribution of Si*NPs prepared by fs laser ablation; (**c**) Schematic presentation of functionalization protocol for the coating of Si*NPs by polyethylene glycol (PEG) and subsequent decoration by radioactive ^188^Re atoms. All images were designed and drawn by authors of this manuscript.

Bare (ligand-free) Si*NPs were fabricated by femtosecond laser ablation in deionized water^11–13^, as shown schematically in Fig. 1a and described in details in the Methods section. Being composed of crystalline Si covered by a 1–2 nm thick oxide shell^13^, laser-synthesized Si*NPs have an ideal spherical shape and are relatively monodispersed, with their mean size being about 25 nm (Fig. 1b). The Si*NPs were coated with PEG according to our newly developed protocol (see methods section), in order to minimize the immune response of the biological system. Due to high hydrophilicity, PEG is known to form a water cloud around the NPs, which protects them from the interaction with antibodies and opsonic proteins, and dramatically increases the circulation of nanomaterials in the blood streem^16^. Finally, we conjugated Si NPs-PEG complex with ^188^Re ions using coordination with the carboxyl group available on the PEG surface, as described in the Methods section.

### Biodistribution of Si*NPs-PEG-^188^Re conjugates under systemic administration

In our tests, biodistribution of the nanoparticle carrier-based Si*NPs-PEG-^188^Re conjugate was compared with that of freely circulating radioactive rhenium using its salt sodium perrhenate form, Na^188^ReO_4_. Five sub-groups of 4 Wistar female rats from the “signal” group, implanted with liver cholangioma RS-1, were intravenously administered with a single dose of 56.8–62.5 µg/kg of animal weight of Si*NPs-PEG-^188^Re conjugates. Similar number of animals from the “control” group were injected with water-dissolved Na^188^ReO_4_, containing radioactive rhenium atoms at the same concentration.

**Figure 2:**
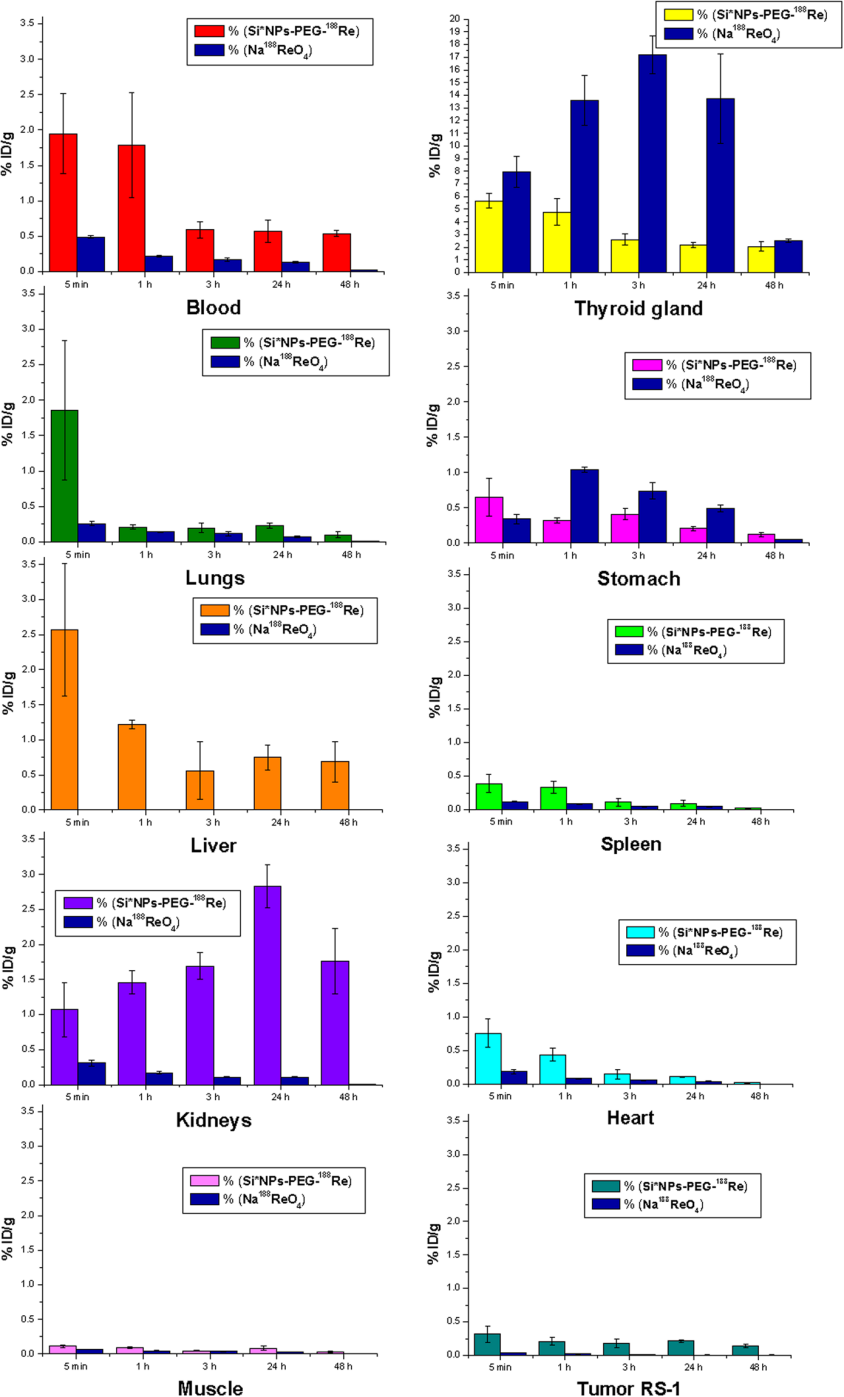
Biodistribution of ^188^Re under its systemic administration in Wistar rats with the nanocarrier-based Si*NPs-PEG-^188^Re conjugate. Different colors show relative amounts of radioactivity for different organs and tissues (blood, thyroid gland, lungs, stomach, liver, spleen, kidneys, heart, muscle, tumor) of Wistar rats with implanted liver cholangioma RS-1 after 5 min, 1 hour, 3 hours, 24 hours and 48 hours of intravenous administration of Si*NPs-PEG-^188^Re complexes. The blue color shows the relative amount of radioactivity in organs and tissues for control group, in which ^188^Re was systemically administered in the free state (with dissolved sodium perrhenate Na^188^ReO_4_ molecules).

As shown in Fig. 2, the maximal level of radioactivity in blood was recorded after 5 minutes of injection of both Si*NPs-PEG-^188^Re and Na^188^ReO_4_ solutions which then gradually decreased. For free ^188^Re (in ^188^ReO_4_^-^), the level of radioactivity in blood was much lower (<0.5%, after 5 min; <0.2% after 1 hour, <0.1% after 24 hours, and finally not detectable after 48 hours). At the same time, the injection of free Na^188^ReO_4_ was accompanied by an immediate increase of the ^188^Re concentration mainly in the thyroid gland, reaching its maximum values of 17% 3 hours after the radionuclide injection. The accumulation of ^188^Re in other organs was much lower (Fig. 2), although we recorded a certain concentration of ^188^Re in the stomach just after the injection (1.2% after one hour), and its smaller concentrations in lungs and kidneys (less than 0.25% and 0.3%, respectively, after five minutes). Notice that the recorded biodistribution and pharmcokinetics with much preferable accumulation of the product in thyroid gland and stomach is typical for free ^188^Re and other radionuclides^1^.

However, the biodistribution and pharmacokinetics were quite different for nanoparticle carrier-based Si*NPs-PEG-^188^Re conjugates. First, the maximal level of ^188^Re in the blood was much higher (1.95% and 1.8% after 5 min and 1 hour, respectively) and easily detectable, even 48 hours after the injection (0.5%). In contrast to the free ^188^Re case, there was no preferential accumulation of radionuclide in any particular organ or tissue. Here, we also recorded certain radionuclide signal in the thyroid gland and stomach (5.5% and 0.65% after 5 min, with a further rapid decrease), but we attributed this signal to washing out of some ^188^Re atoms from the Si*NPs-PEG-^188^Re complexes due to possible non-optimized protocol of their conjugation. Surprisingly, the accumulation of radionuclide in liver and spleen was very weak (less than 2.5% and 0.3% after 5 minutes and then rapidly decreased down to 0.5–0.7% and 0.05% after 24 hours, respectively). The absence of any significant accumulation of Si*NPs-PEG-^188^Re conjugates in organs of reticuloendothelial system (liver, spleen) can only be explained by their invisibility to the immune system, which was obviously due to the PEG-based coating of Si*NPs. As follows from Fig. 2, such a coating led to prolonged circulation of Si*NPs-PEG-^188^Re conjugates in the blood stream and their efficient delivery to most organs. It is also important that the concentration of ^188^Re gradually increased in the kidneys, reaching its maximal value 24 hours after the injection (almost 3%), which is consistent with gradual dissolution of nanoformulations and their time-delayed elimination via renal clearance^12,13^. For comparison, in the case of free rhenium (injection of Na^188^ReO_4_ solutions) its concentration in the kidney was maximal just after the injection (5 min), which can lead to undesirable kidneys damage.

### Biodistribution of Si*NPs-PEG-^188^Re conjugates under intratumoral administration

**Figure 3:**
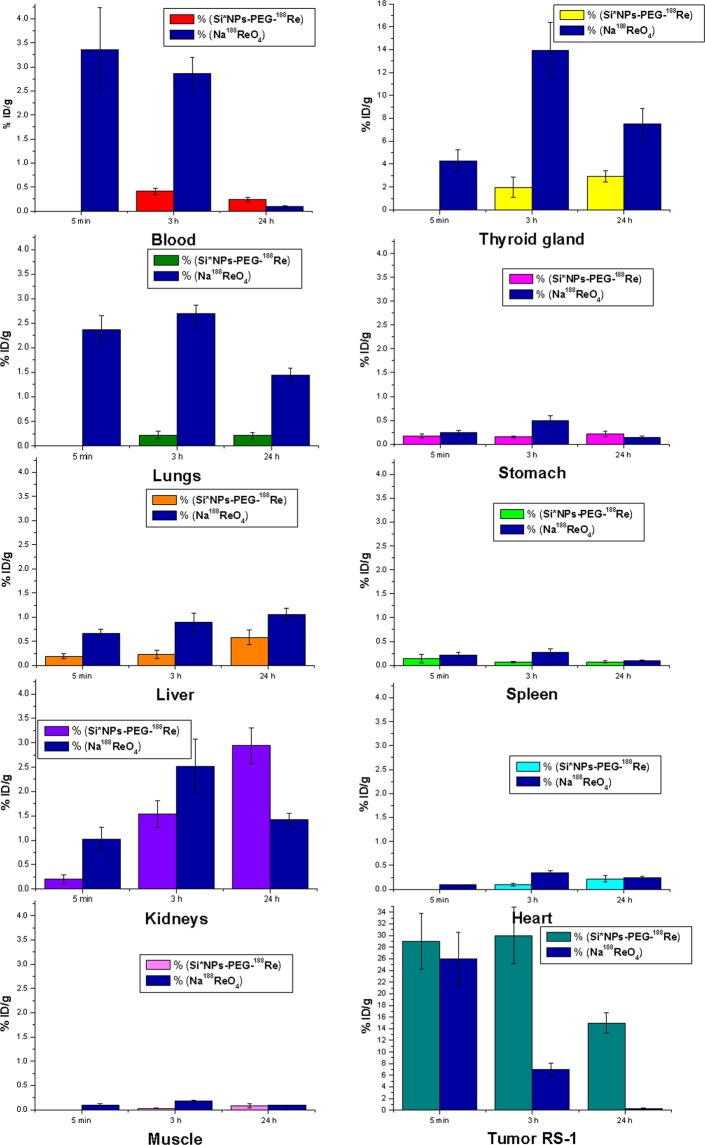
Biodistribution of ^188^Re under its intratumoral administration with the nanocarrier-based Si*NPs-PEG-^188^Re conjugate. Different colors show relative amount of radioactivity in the organs and tissues of Wistar rats (blood, thyroid gland, lungs, stomach, liver, spleen, kidneys, heart, muscle, tumor) with implanted liver cholangioma RS-1 after 5 min, 3 hours and 24 hours of intratumoral administration of the Si*NPs-PEG-^188^Re complexes.The blue color shows relative amount of radioactivity in organs and tissues for control group subjected to intratumoral injection of Na^188^ReO_4_.

Three sub-groups of 4 Wistar rats with implanted cholangioma RS-1 from the “signal” group were intratumorally administered with a single dose of 56.8–62.5 µg/kg of nanoparticle carrier-based Si*NPs-PEG-^188^Re complexes, while the same number of animals from the “control” group were intratumorally administered Na^188^ReO_4_ solutions having a similar concentration of ^188^Re atoms. Different sub-groups of animals from the “signal” and the “control” groups were sacrificed 5 minutes, 3 hours and 24 hours after the injection and examined for ^188^Re distribution in different organs. As shown in Fig. 3, in the case of free ^188^Re atoms, we recorded a drastic (4-fold) decrease of ^188^Re concentration in the tumor during the first 3 hours (from 25% to 6%), while after 24 hours, it was not detectable in this area, suggesting a fast washing out of the radionuclide. At the same time, we recorded a fast increase of ^188^Re concentration in blood (3.75% after 5 min),with a further slow decrease down to 2.75% after 3 hours and 0.05% after 24 hours. After 3 hours, ^188^Re mostly migrated into the thyroid gland (14%) that looks consistent with typical biodistribution for this radionuclide. Significant concentrations of ^188^Re were also recorded in lungs, kidneys and liver (2.75%, 2.5% and 1% after 3 hours, respectively), while its concentration in the stomach was much lower compared with intravenous injection (0.4% after 3 hours). In general, our data on intratumoral injection of free ^188^Re showed immediate washing out of the radionuclide from the tumor area and its further accumulation preferably in the thyroid gland. As shown in Fig. 3, nanoparticle carrier-based Si*NPs-PEG-^188^Re conjugate demonstrated a radically different biodistribution and pharmacokinetics. Here, we did not observe any decrease of the ^188^Re concentration in the tumor during the first 3 hours (its value was always higher than 30%) and the concentration of the radionuclide in this area was very high (>15%) even after 24 hours. Thus, due to the employment of Si*NPs-based carrier, we had very good retention of ^188^Re over its half-decay time, enabling maximal therapeutic effect. On the other hand, the migration of ^188^Re to other organs was very weak, although we recorded certain accumulation of the radionuclude in the thyroid gland (less than 2.8%), blood (less than 0.3%), lungs (less than 0.2%), liver (less than 0.6%), stomach (less than 0.15%) and spleen (less than 0.1%). We believe that a relatively strong signal in the thyroid gland could arise from washing out of some ^188^Re atoms from Si*NPs-PEG-^188^Re conjugates, similarly to what happened after intravenous injection, while the increase of ^188^Re concentration in other organs can be due to the interjection of certain number of Si*NPs-PEG-^188^Re conjugates from the tumor to the blood stream. Of particular attention, we can mention a gradual increase of ^188^Re concentration in the kidneys, with a maximal value of 3% reached 24 hours after the injection, which contrasts the data for free rhenium atoms. To summarize, our tests established a very good retention of ^188^Re in the tumor, which shows promise for successful use of Si*NPs as carriers of radionuclides.

### Therapeutic efficiency using Si*NPs-^188^Re conjugates

**Figure 4:**
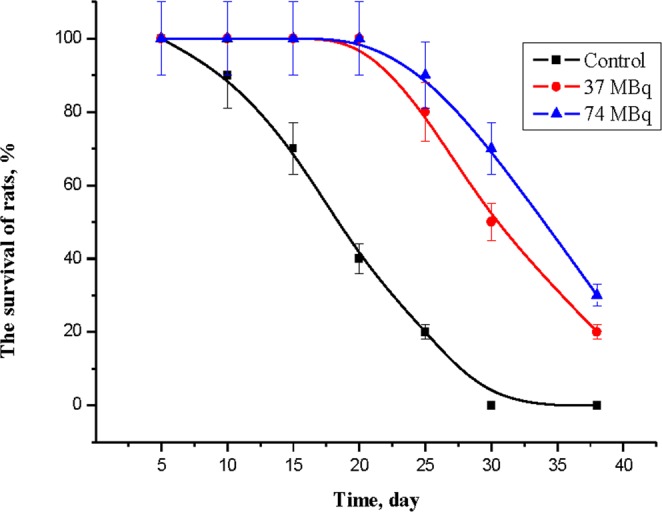
Assessment of therapeutic effect. Survival curves for Wistar rats with implanted cholangioma RS-1 after intratumoral injection of the Si*NPs-PEG-^188^Re conjugates providing different doses of radioactivity (37 and 74 MBq) and for control group injected with physiological solutions. Each group was composed of 10 animals.

The therapeutic efficiency of Si*NPs-^188^Re conjugates was assessed by using Wistar rats with cholangioma RS-1 implanted in the right femoral muscle. We used 30 rats divided into three sub-groups of 10 animals: the 1^st^ and 2^nd^ “signal” groups were intratumorally administered with a single dose of 37 and 74 MBq of NPs carrier-based Si*NPs-PEG-^188^Re conjugates, respectively, while the 3^rd^ “control” group were intratumorally injected by 0.1 mL of physiological solutions. Figure 4 shows results of survival tests for these 3 groups. One can see that after 20 days, only 40% of rats from the control group survived, while the survival rate for the 1^st^ and 2^nd^ “signal” groups was 100%. After 30 days, all animals from the control group were dead, while the survival rate for the 1^st^ and 2^nd^ groups was 50% and 72%, respectively. Thus, our experiments clearly demonstrate a remarkable therapeutic effect under intratumoral injection of the Si*NPs-PEG-^188^Re conjugates. It should be noted that the accomplished injection protocol was not optimized to maximize the therapeutic effect. We believe that the efficiency of the treatment can still be improved, e.g., by using 2 and more injections and further optimization of dose radioactivity.

### Biodegradation, bioelimination and safety of conjugates

As shown in Fig. 2, intravenous administration with the Si*NPs-PEG-^188^Re conjugates leads to at least a 10-fold higher concentration of ^188^Re in the kidneys compared to the case of free ^188^Re atoms (injection of Na^188^ReO_4_ solutions). This unambiguously indicates that the radionuclide comes to the kidneys in the conjugated state. The pharmacokinetics of ^188^Re is also completely different in the case of the Si*NPs-PEG-^188^Re conjugates, as the radionuclide comes not immediately, but after some delay (24 hours).

It should be noted that by themselves, the Si*NPs prepared by laser ablation present a highly safe product for biomedical use, as follows from the results of our recent tests in a mouse model^12,17^. Here, we considered the worst “stress” scenario, when the NPs are bare (non-PEGylated) and should be immediately sequestrated by the reticuloendothelial system. Indeed, after systemic administration, almost 100% of Si*NPs immediately accumulated in the liver and the spleen, but in contrast to silica (SiO_2_) and many other nanomaterials whose accumulation in the liver causes a series of damaging effects (hyperplasia of Kupffer cells, hepatic inflammation, oxidative stress etc.^18^), we observed only minor inflammation effects which completely disappeared 48 h after the injection, as evidenced by a histopathological investigation of tissues^12^. At the same time, we recorded stability of blood parameters (aminotrasferases, alkaline phosphatase, bilirubin, cholesterol, etc.)^17^ and absence of any liver or kidney toxicity, as was confirmed by ALAT, ASAT and the serum creatinine levels, and negligible changes of oxidative stress parameters including catalase, SOD, GPx activities, Vit A and E72^12^. Furthermore, Si*NPs started to decompose into orthosilicic acid *Si(OH)*_4_ soon after the administration and then migrated to kidneys where the decomposition process continued to reduce the NPs size down to a renal glomerular filtration range (<7 nm), rendering possible their excretion with the urine. The complete bioelimination process took 5–7 days, as was controlled by monitoring the Si content in the urine^12^. In the presented study, Si*NPs were additionally PEGylated, which prolonged the circulation time in the organism and radically changed biodistribution, giving access to most organs. Nevertheless, the Si*NPs-PEG-^188^Re conjugates similarly migrated to kidneys where they were supposed to decompose and finally excrete via renal filtration. It is important that such filtration starts only after some delay (typically, after 24 hours), which should minimize damaging effects in the kidney as the radioactivity of ^188^Re is already much lower after the half-decay time.

To summarize, we established the merits of the Si*NPs as safe and effective carriers of ^188^Re radionuclide for nuclear therapy. Our study has revealed a quite different biodistribution and pharmacokinetics of the Si*NPs-PEG-^188^Re conjugates compared to the free ^188^Re atoms (water-dissolved sodium perrhenate Na^188^ReO_4_) in a Wistar rat model. Our tests on intravenous administration showed that the NPs-based carrier conjugate can freely circulate in the blood stream and target tumors, while the free ^188^Re atoms mostly accumulate in the thyroid gland. In addition, intratumoral administration tests evidenced very good retention of the radionuclide in the tumor for more than 24 hours, while the free ^188^Re rapidly washed out form the tumor under similar conditions. Under both administrations, we recorded at least a 24-hour delayed delivery of ^188^Re to kidney for the Si*NPs-PEG-^188^Re conjugates, which is consistent with gradual decomposition of these complexes; it promises much reduced side effects in the kidneys. Finally, our tests evidenced a considerable increase of the rat survival rate for the groups of animals intratumorally administered with the Si*NPs-PEG-^188^Re conjugates; the radioactive doses of the Si*NPs-PEG-^188^Re conjugates compared to the control group were: (i) 100% for both groups compared to 40% after 20 days; (ii) 50% and 72% compared to 0% after 30 days.

We believe that the Si*NPs-based transport vehicle complex can be considered as a general biodegradable platform for targeted delivery of radionuclides for nuclear therapy. We demonstrated its successful conjugation with ^188^Re, which is one of the very efficient beta-emitters that can be synthesized in portable ^188^W/^188^Re generators, making possible its low-cost fabrication and worldwide distribution^19^. Nevertheless, other promising diagnostic (^64^Cu, ^68^Ga) or therapy (e.g., ^90^Y) radionuclides can equally be conjugated with biodegradable Si*NPs carriers to maximize the efficacy of imaging or therapy. It is also important that crystalline nano-Si is a IV group semiconductorwhose intrinsic properties make possible a series of unique imaging and therapy functionalities, including room temperature photoluminescence for bioimaging^20–22^, light-induced generation of singlet oxygen for photodynamic therapy of cancer^23^, and infrared^24^, radio frequency^17^, and ultrasound-induced^25^ cancer hyperthermia. In fact, the choice of Si*NPs as radionuclide carrier means that all these imaging and therapy modalities can be utilized in parallel with the main nuclear medicine modality to produce image guided therapy, and thus maximize the final therapeutic outcome. One of the most promising tandem therapeutic approaches, we see, is radio frequency-induced hyperthermia using Si*NPs as sensitizers of local heating^17^ which can be used even for the treatment of deep tissues due to good transparency of the body to the RF radiation. As another additional functionality, one can imagine the use of fluorescence properties of Si*NPs to track the localization of therapeutic agents in the tumor, although this modality is limited by superficial tissues due to low transmission of light even in the spectral range of relative tissue transparency (750–900 nm).

## Methods

### Synthesis and characterization of Si*NPs

Si*NPs were prepared by ultra-short (fs) laser fragmentation in water ambience, as described in our recent publications^11,13^. Briefly, a powder of 0.5 µm Si microparticles, preliminarily prepared by mechanical milling of a Si wafer, was introduced into a glass cuvette at 0.35 g/L and dispersed in deionized water by a sonication bath step for 30 minutes. The dispersed Si microparticles were then fragmented under laser irradiation for one hour using a Yb:KGW (fs) laser (Avesta Inc., Russia, 1030 nm, 270 fs, 1–30 kHz). The laser beam was focused at 1 cm below the water level, while the solution was continually homogenized by a magnetic stirrer. In addition, the initial concentration was varied in the range 0.15 g/L to 0.5 g/L in order to control the mean size of the NPs according to the protocol proposed in ref.^11^. To determine the size characteristics of nanoparticles, a high-resolution transmission electron microscopy (HR-TEM) system (JEOL JEM 3010) was employed in the imaging and diffraction modes. A droplet of solution containing laser-synthesized nanoparticles was deposited onto the surface of a carbon-coated TEM copper grid, dried and finally examined by the TEM system.

### Chemical modification and functionalization of Si*NPs

#### Materials

Silane-PEG-COOH (average Mw 5000) were purchased from Biochempeg Scientific Inc. Ethanol and 30% ammonium hydroxide were obtained from Sigma-Aldrich. MilliQ-grade water was used in the preparation of buffers and aqueous solutions.

#### PEG-based coating of Si*NPs

The functionalization of laser-synthesized Si*NPs with polyethylene glycol was performed as follows. The Si*NPs were dispersed in 10 mL of 96% ethanol to a final concentration of 2 g/L. Then, 200 mg of the silane-PEG-COOH solution in 20 mL of ethanol was added to the NPs under continuous stirring at room temperature. Since only dense coating of the NPs by PEG is able to provide stealth properties, we used a large molar excess of the silane-PEG chains in this reaction. The resulting mixture was ultra-sonicated for 1 min, and 1 mL of 3% ammonium hydroxide was quickly drop added into the mixture under vigorous stirring to catalyze the hydrolysis and condensation of the silane groups on the surface of the Si*NPs. Then we tested if the pH of the mixture had reached 9–10, and heated this solution for 2.5 h at 70 °C. To prevent further hydrolysis and self-aggregation of unreacted silanes, the mixture was cooled down to the room temperature and the NPs were washed by centrifugation (15 min, 5000 g), firstly with pure ethanol and further with water 3 times. After washing, the product was redispersed in 20 mL of PBS (pH 7.4). The obtained NPs suspension did not contain any aggregates and had long-term colloidal stability at physiological conditions, as was confirmed by Dynamic Light Scattering (Zetasizer Nano ZS, Malvern Instruments, UK). Also, we observed a strong negative zeta-potential after coupling of the PEG-COOH chains with the silicon surface. All the measurements were conducted in MilliQ water.

### Preparation of the Si*NPs-PEG-^188^Re conjugates

#### Preparation of radioactive ^188^Re solutions

^188^Re was obtained in the form of a Na^188^ReO_4_ solution by elution with saline from a column of a ^188^Re/^188^W generator (A. I. Leipunsky Institute of Physics and Power Engineering, Joint Stock Company, Obninsk, Russia). The radioactive purity of the ^188^Re eluate exceeded 99%. Volume activity of eluate ^188^Re was 185 MBq·ml^−1^ (5,0 mCi·ml^−1^).

#### Conjugation of ^188^Re with Si*NPs carrier and assessment of its efficiency

The conjugation was performed according to the protocol developed in ref.^26^. Briefly, 5 ml of the Si*NPs-PEG complexes having a concentration of 1 mg/ml was added to 1.5 ml of distilled water and 7 mg of ascorbic acid. The ingredients were then mixed for 5 min via ultrasound, to which was added 9.5 mg of SnCl_2_ 2H_2_O (5.0 mg for Sn^2+^) in 0.1 ml 0.1 M HCl. After 5-minutes of mixing by ultrasound for 5 min, the 74 MBq ^188^Re eluate in 0.2 ml of physiological solution was added to it and then mixed once again for 1 hour. Then, a centrifugation step was applied to wash out the unconjugated ^188^Re elute. Then, the superficial layer was removed, while the precipitate was re-suspended in 4 ml of physiological solution. The centrifugation procedure was repeated once again to remove the superficial layer. The precipitate was once again re-suspended in 2 ml physiological solution and examined for radiochemical purity using chromatography methods. The efficiency of conjugation of ^188^Re with the Si*NPs-PEG complex was determined by paper chromatography using a Whatman 1 paper (Germany). 3.0 µl samples of the reaction mixture were applied with a micropipette onto chromatographic paper stripes (10*110 mm). The stripes were placed vertically in a beaker, and elution was performed with acetone buffer. The Si*NPs-PEG-^188^Re conjugate stayed at the bottom start region of the stripes (R_f_ = 0.1–0.15), while the free ^188^ReO_4_^−^ ions ascended with the eluent front, until R_f_ = 0.8–0.9. The amounts of the Si*NPs-PEG-^188^Re and free ^188^Re were determined by radiometry of chromatographic paper stripes using an automatic gamma counter, “Wizard 2480” (Perkin Elmer/Wallac, Finland). Our tests showed that the radiometric outcome of Si*NPs-PEG-^188^Re was 59.2 MBq in 2 ml (29.6 MBq/ml), which corresponds to 80% of the initial radioactivity of the ^188^Re eluate (Supplementary Fig. 1Sa). We also found that the ^188^Re-based nanoconjugates were stable for more than 48 hours, while the impact of radionuclide impurities after the washing procedure was less than 5% (Supplementary Fig. 1Sb).

### Methodology of animal tests

#### Implantation of RS-1 tumor

All experiments were carried out using female Wistar rats with a body weight of 120–140 g (branch of “Stolbovaya” of the Scientific Center of Biomedical Technologies of Federal Medical-Biological Agency (FMBA)). As a tumor model, we used cholangioma RS-1 (Tumor strain depository of N.N. Blokhin National Medical Research Center of Oncology of the Ministry of Health of the Russian Federation). To obtain an initial sample of the solid tumor, a donor rat with the tumor was sacrificed and the tumor tissue was extracted. Then, the tumor tissue was fragmented, diluted in physiological solution in the proportion of 1:3, and implanted into the right femoral muscle. Each injection was about 100 µg of the tissue in 0.1 ml per animal, which was optimal for cholangioma RS-1, as it ensured 100% implantation of the tumor and its good growth, as well as provided maximal lifetime for the animals. Every three days, the tumor volume of every animal was examined and its volume was assessed. 8–10 days after the implantation, when the tumor volume was about 0.7–0.8 cm^3^, all animals were subdivided into “signal” and control groups, according to the planned experiments.

#### Intravenous administration of Si*NPs-PEG-^188^Re and free ^188^Re

40 Wistar rats were divided into 2 groups (20 rats in each). Rats from the first group were intravenously injected in the jugular vein (under isofluorane anesthesia) with a single dose of 0.74–1.11 MBq of Si*NPs-PEG-^188^*Re* in 0.1 ml of physiological solution (12.5 µg of Si*NPs-PEG-^188^*Re)*, which provided 56.8–62.5 µg per kg of animal weight. Rats from the second group were injected in the jugular vein with a single dose of 0.74–1.11 MBq of free Na^188^ReO_4_) in 0.1 ml of physiological solution, which provided 3.7–5.55 MBq per kg of animal weight.

#### Intratumoral administration of Si*NPs-PEG-^188^Re and free ^188^Re

24 Wistar rats were divided into 2 groups (12 rats in each). Rats from the first group were intratumorally (in the tumor center) injected with a single dose of 0.74–1.11 MBq of Si*NPs-PEG-^188^*Re* in 0.1 ml of physiological solution (12.5 µg of Si*NPs-PEG-^188^*Re)*, which provided 56.8–62.5 µg per kg of animal weight. Rats from the second group were intratumorally injected in a similar way with a single dose of 0.74–1.11 MBq of free Na^188^ReO_4_) in 0.1 ml of physiological solution, which provided 3.7–5.55 MBq per kg of animal weight.

#### Methodology of ^188^Re biodistribution experiments

Different sub-groups of animals (4 of each) were sacrificed 5 minutes, 1 hour, 3 hours, 24 hours and 48 hours after the injection of both solutions. Samples of key organs and tissues were collected, weighted in electronic balance (“Sartorius”, Germany) and placed in plastic boxes to assess the intensity of ionizing emission from ^188^Re by a gamma counter (2480 Wizard, Perkin Elmer-Wallac, Finland). 0.1 mL of the Si*NPs-PEG-^188^Re solution was collected at the moment of NPs administration in mice, and placed in a separate cuvette to serve as a calibration standard. Based on the radiometric data for every observation point, we calculated the relative radioactivity per 1 g of organs or tissues as well as the total radioactivity of all organs or tissues.

All experimental procedures and animal care were carried out in accordance with the legislation of Russian Federation, directive 2010/63/EU of European Parliament and EU Council from 22 September 2010, as well as with the National Institutes of Health Guide for the Care and Use of Laboratory Animals (NIH Publ. No. 80–23, revised 1996). Experiments and animal care were performed at the National Medical Research Radiological Center (NMRRC) of the Ministry of Health of the Russian Federation, Obninsk, Russia. All experimental protocols were approved by the Scientific Council and Committee of Ethics on Animal experiments of NMRRC.

## Supplementary information

Supplementary Information
