# Supplementary material for: Nuclear nanomedicine using Si nanoparticles as safe and effective carriers of 188Re radionuclide for cancer therapy
Source: Sci Rep. 2019 Feb 14;9:2017. doi: 10.1038/s41598-018-38474-7 (PMC6376125; doi:10.1038/s41598-018-38474-7)
Supplement: Supplementary file 1 — Supplementary Information [file 41598_2018_38474_MOESM1_ESM.docx]

**Supplementary Information**

**Nuclear nanomedicine using Si nanoparticles as safe and effective carriers of ^188^Re radionuclide for cancer therapy**

V. M. Petriev^1,2^, V. K. Tischenko^2^, A. A. Mikhailovskaya^2^, A. A. Popov^3^, G. Tselikov^3^, I. Zelepukin^4^, S. M. Deyev^1,4^, A. D. Kaprin^2^, S. Ivanov^2^, V. Yu. Timoshenko^1,5^, P. N. Prasad^1,6*^, I. N. Zavestovskaya^1^, A. V. Kabashin^1,3*^

^1^MEPhI, Institute of Engineering Physics for Biomedicine (PhysBio), 115409 Moscow, Russia

^2^National Medical Research Radiological Center of the Ministry of Health of the Russian Federation, Obninsk, Russia

^3^Aix Marseille Univ, CNRS, LP3, Campus de Luminy – Case 917, 13288, Marseille, France

^4^Shemyakin–Ovchinnikov Institute of Bioorganic Chemistry, Russian Academy of Sciences, 16/10 Miklukho-Maklaya St, Moscow 117997, Russia

^5^Lomonosov Moscow State University, Physics Department, Leninskie Gory 1, 119991, Moscow, Russia

^6^Department of Chemistry and Institute for Lasers, Photonics, and Biophotonics, University at Buffalo, The State University of New York, Buffalo, New York 14260, United States.

**Chromatography data to determine efficiency and stability of Si*NPs-PEG-^188^Re complex**

**(a)**

**(b)**

**Figure 1S.** (a) Dynamics of conjugation of ^188^Re with the Si*NPs-PEG complex during 48 hours; (b) Temporal stability of the ^188^Re-based nano-conjugates on Whatman paper after washing out the unconjugated ^188^Re eluate

As shown in Fig. 1Sa, the efficiency of conjugation is about 80% related to initial radioactivity of ^188^Re eluate. As follows from Fig. 1Sb, the Si*NPs-PEG-^188^Re conjugates were stable during first 48 hours, while the impact of radionuclide impurities after washing out unconjugated ^188^Re eluate was lower than 5%.
